# Endoplasmic Reticulum-Mitochondria Contacts: A Potential Therapy Target for Cardiovascular Remodeling-Associated Diseases

**DOI:** 10.3389/fcell.2021.774989

**Published:** 2021-11-10

**Authors:** Yu Wang, Xinrong Zhang, Ya Wen, Sixuan Li, Xiaohui Lu, Ran Xu, Chao Li

**Affiliations:** ^1^Innovation Research Institute of Traditional Chinese Medicine, Shandong University of Traditional Chinese Medicine, Jinan, China; ^2^Emergency Department, Affiliated Hospital of Shandong University of Traditional Chinese Medicine, Jinan, China; ^3^Jinan Tianqiao People’s Hospital, Jinan, China

**Keywords:** endoplasmic reticulum, mitochondria, cardiovascular remodeling, calcium transfer, therapeutic strategies

## Abstract

Cardiovascular remodeling occurs in cardiomyocytes, collagen meshes, and vascular beds in the progress of cardiac insufficiency caused by a variety of cardiac diseases such as chronic ischemic heart disease, chronic overload heart disease, myocarditis, and myocardial infarction. The morphological changes that occur as a result of remodeling are the critical pathological basis for the occurrence and development of serious diseases and also determine morbidity and mortality. Therefore, the inhibition of remodeling is an important approach to prevent and treat heart failure and other related diseases. The endoplasmic reticulum (ER) and mitochondria are tightly linked by ER-mitochondria contacts (ERMCs). ERMCs play a vital role in different signaling pathways and provide a satisfactory structural platform for the ER and mitochondria to interact and maintain the normal function of cells, mainly by involving various cellular life processes such as lipid metabolism, calcium homeostasis, mitochondrial function, ER stress, and autophagy. Studies have shown that abnormal ERMCs may promote the occurrence and development of remodeling and participate in the formation of a variety of cardiovascular remodeling-associated diseases. This review focuses on the structure and function of the ERMCs, and the potential mechanism of ERMCs involved in cardiovascular remodeling, indicating that ERMCs may be a potential target for new therapeutic strategies against cardiovascular remodeling-induced diseases.

## Introduction

With an aging society and the change in people’s lifestyles, on a global scale, the incidence and mortality of cardiovascular disease (CVD) have been increasing for decades (Bansilal et al., 2015). In 2019, there were nearly 523 million new cases of cardiovascular disease and 18.6 million deaths from cardiovascular disease worldwide (Andersson and Vasan, 2018; Roth et al., 2020). The World Health Organization (WHO) has classified CVD, as well as cancer and rheumatic immune diseases (Capewell and Lloyd-Jones, 2010), as a threat to human health.

Cardiovascular remodeling plays a crucial role in the pathological process of CVD (Porter and Turner, 2009), and it constitutes a pathological change in the structure and function of cardiovascular tissue produced under a series of physiological stimuli (such as exercise or pregnancy) and pathological stimuli (such as hypertension, diabetes, or myocardial infarction) (Sekaran et al., 2017). The molecular mechanisms involved in cardiovascular remodeling are multi-angular and multi-dimensional, and include various inflammatory pathways and other molecular biological dimensions (Martinez-Martinez et al., 2015), autophagy mechanisms (Shi et al., 2021; Sciarretta et al., 2018a), angiogenesis mechanisms (Mentzer and Konerding, 2014; Saraf et al., 2016), and gene transcription regulation (Van Guilder et al., 2021; Zhang C. et al., 2021). Moreover, in abnormal cellular energy metabolism (Akhmedov et al., 2015; Kimball and Vondriska, 2020), oxidative stress (Münzel et al., 2017), autophagy defects (Sciarretta et al., 2018b), calcium homeostasis imbalance (Chaanine et al., 2013), ER stress (Lebeau et al., 2018), and apoptotic activation (Intengan and Schiffrin, 2001), remodeling may occur (Yang Y. et al., 2019). An increasing number of studies has begun to focus on cardiovascular remodeling from the overall functional level of organelles and organelle interaction, especially the mitochondria (Nunnari and Suomalainen, 2012) and ER/sarcoplasmic reticulum (SR) (Reddish et al., 2017). ERMCs are architecture and ultrastructural organization that exist between mitochondria and the ER which involve various cellular life processes (Szymański et al., 2017). Accumulating evidences (Zhou et al., 2018a; Gao et al., 2020; Silva-Palacios et al., 2020; Yang et al., 2020) has demonstrated that abnormal ERMCs may be pivotal in the development of cardiovascular remodeling because they are involved in the development of a variety of cardiovascular remodeling-related diseases.

In recent years, researchers have found that during their physiological functions, mitochondria and the ER are not isolated and do not act divisively (Gomez-Suaga et al., 2017b). On the contrary, there is a very close relationship between mitochondria and the ER in structure and function (Kornmann, 2013). They jointly exercise the responsibility of maintaining the stability of the intracellular environment (Lee and Min, 2018). In the past decade, there has been an explosive growth in knowledge regarding the role of ERMCs in cardiovascular remodeling (Lee and Min, 2018; Vannuvel et al., 2013), and clinical studies have found that energy-intensive and calcium signal-regulated dependent cells such as cardiomyocytes are very susceptible to mitochondrial and ER dysfunction, especially ERMCs (Kho et al., 2012; Chan, 2020). Mitochondria are the main productive organelles and the second largest calcium pool in eukaryotic cells (Ruan et al., 2020). They are also multifunctional and dynamically plastic organelles (Kummer and Ban, 2021) that are involved in various biological processes such as steroid synthesis (Chow et al., 2017), lipid metabolism (Benador et al., 2019), calcium signal transduction (De Stefani et al., 2016) and apoptosis (Bhola and Letai, 2016). As the largest intracellular calcium store (Raffaello et al., 2016), the ER is a multifunctional organelle that is the main site of protein synthesis, folding, transport (Guerriero and Brodsky, 2012), calcium homeostasis regulation (Stutzmann and Mattson, 2011) and lipid biosynthesis (Yousuf et al., 2020). The ER has the most extensive intracellular membrane structure and forms structural and functional coupling regions with a variety of intracellular organelles (Wang and Kaufman, 2016), of which the most important coupling organelle is the mitochondria, and their binding sites are known as mitochondria-associated ER membranes (MAMs) (Cheng et al., 2020). ERMCs and MAMs are sometime used as synonyms, but they do not mean the same thing. ERMCs are architecture and ultrastructural organization that exist between mitochondria and the ER and involve various cellular life processes (Giacomello and Pellegrini, 2016; Szymański et al., 2017). The term MAMs, instead, are the product of the biophysical enrichment of mitochondria and ER membranes tethered together, and describes the repertoire of proteins and lipids that form the ERMCs (Giacomello and Pellegrini, 2016; Szymański et al., 2017). MAMs are the binding sites and structural basis of ERMCs. In some papers, ERMCs were also written as “mitochondria-endoplasmic reticulum contacts (MERCs) (Giacomello and Pellegrini, 2016; Cheng et al., 2020).

Under physiological conditions, mitochondria and the ER are physiologically interconnected through MAMs to participate in basic cell biological processes including lipid (Tatsuta et al., 2014) and calcium (Ca^2+^) homeostasis (Bagur and Hajnóczky, 2017), mitochondrial dynamics (Klecker et al., 2014), and other related cellular behaviors such as autophagy (Senft and Ronai, 2015), ER stress (Murphy, 2013), inflammation (Roca et al., 2019), and apoptosis (Rosati et al., 2010). The complex functional regulatory structure is involved in the development of various cardiovascular diseases, including cardiac hypertrophy (Tuncay et al., 2019), heart failure (Liu et al., 2014), metabolic cardiomyopathy (Liu et al., 2014), ischemic heart disease (Li et al., 2016), and arrhythmia (Silva-Palacios et al., 2020).

Dysfunction of ERMCs is involved in the homeostasis reconstruction of cardiomyocytes (Chen J. et al., 2021), protection against oxidative stress (Grings et al., 2019), Ca^2+^ signaling (Bagur and Hajnóczky, 2017), lipid metabolism (Biczo et al., 2018), energy metabolism (Szymański et al., 2017), and cell survival (Zhou et al., 2018a). Therefore, in this review, we will summarize the structure and function of the ERMCs, and then we will discuss how ERMCs are associated with the high prevalence of cardiovascular diseases such as cardiac hypertrophy, heart failure, and systemic vascular diseases, as well as the potential use of ERMCs as therapeutic targets for cardiovascular remodeling.

## Structure of Endoplasmic Reticulum-Mitochondria Contacts

### Formation of Mitochondria-Associated Endoplasmic Reticulum Membranes

In 1956, an intimate relationship between mitochondria and ergastoplasm was discovered in rat liver cells (Bernhard and Rouiller, 1956). In 1990, MAMs were interpreted as a point of contact between ER membranes and mitochondria by Vance (1990). Electron tomography has shown that the ER and mitochondria are connected by tethers (9–16 nm for smooth ER and 19–30 nm for rough ER) (Simmen and Herrera-Cruz, 2018; Zhao et al., 2020). Further studies showed that MAMs are formed when the ER is close to the mitochondria by approximately 20 nm on 20% of the mitochondrial surface (Csordas et al., 2006). The presence of MAMs promotes communication between the ER and mitochondria, and also endows both with new properties and functions. MAMs are structurally heterogeneous, and according to the size of the ER and mitochondrial contact areas observed under electron microscopy, MAMs can be divided into three types: in type I, the contact area between the ER and mitochondria is approximately 10%, and in the case of mitochondrial elongation, only a portion of the mitochondria is anchored to the ER, and the rest can move independently. Most human intracellular MAMs are of this type. In type II, the contact area between the ER and mitochondria is approximately 50%, and this type is often present in brown adipose tissue. In type III, the ER completely covers the mitochondria (Fujimoto and Hayashi, 2011).

Proteomics indicate the presence of specific molecules in specific sites on ERs that form different ER domains, which is also one of the sources of structural heterogeneity of MAMs (Xu H. et al., 2020). The ER has a universal pluripotent fusion property, leading to active formation of physical contacts between two different membranes (Nguyen et al., 2020). Active extension of ER protrusions to other organelles is frequently observed (Gagnon et al., 2002). The ER may facilitate interactions with mitochondria if proteins necessary for membrane association are present on both sides. Thus, as the ER establishes a large monolayer membrane network, ERMCs may occur during ER maturation (Nam and Jeon, 2019). Moreover, the trafficking between the ER and Golgi apparatus appears to be pivotal and the structural integrity of the ER and the normal ER-Golgi vesicle transport function are essential for the structural maintenance of mitochondria and their formation with MAMs (Coutinho et al., 2004).

Recent studies have shown that the regulation of ERMCs and mitochondrial dynamics are two closely intertwined processes (Friedman et al., 2011). Studies have shown that the sites of MAMs in rat heart cells coincide with the outer mitochondrial membrane (OMM) – inner mitochondrial membrane (IMM) contact sites, and therefore, MAMs may form calcium channel complexes to regulate Ca^2+^ transport between the endoplasmic reticulum and mitochondria (Jin et al., 2021). The inositol 1,4,5-triphosphate receptor (IP3R) is located in the ER membrane and regulates Ca^2+^ release, and voltage-dependent anion selective channel protein (VDAC) 1 is a Ca^2+^ uptake channel on the OMM (Szabadkai et al., 2006). Similarly, another important Ca^2+^ release channel, the ryanodine receptor (RyR), is also expressed at MAMs and plays a key role in Ca^2+^ transport (Jakob et al., 2014; Yang et al., 2018).

Proteins involved in mitochondrial fusion also regulate ER morphology and MAMs. Mitochondrial fusion protein 2 (Mfn2) is located on the surface of the OMM, connecting adjacent mitochondria, and also on the surface of the ER, which can form dimers with Mfn1 or Mfn2 located on the surface of mitochondria, thereby connecting the ER to mitochondria and promoting mitochondrial Ca^2+^ transport (de Brito and Scorrano, 2008). Silencing Mfn2 in mouse embryonic fibroblasts and HeLa cells not only disrupts the ER, but it can also alter the normal structure of MAMs (Wang et al., 2012).

### Composition of Mitochondria-Associated Endoplasmic Reticulum Membranes

A series of proteins constitutes the tether of MAMs, which are expressed on the ER membrane and the OMM, thus connecting the two organelles. The protein components on MAMs are classified according to the functions performed by the protein complexes (Li et al., 2020a): (1) Ca^2+^ regulatory proteins, such as IP3Rs/GRP75/VDAC1 complexes and Sigma-1 receptors (Sig1Rs); (2) lipid synthesis and transport proteins; (3) MAM tethers proteins, such as VAPB-PTPIP51; (4) MAM controlled proteins, such as phosphofurin acidic cluster sorting protein-2 (PACS-2) and Mfn2. Depletion of certain proteins or interruption of certain protein-protein interactions on MAMs can lead to disruption of the structure and function of MAMs (as shown in Table 1).

**TABLE 1:** Summary of the functional proteins at mitochondria-associated endoplasmic reticulum membranes (MAMs).

Components	Roles	Interactions	Functions and mechanisms	References
**(1) Ca^2+^ regulatory proteins**				
IP3Rs	ER-resident Ca^2+^ receptors	Forming IP3Rs/GRP75/VDAC1 complex	Interaction with GRP75 and VDAC1, promoting Ca^2+^ release from ER in MAMs	Bartok et al., 2019
GRP75	Cytosolic molecular chaperone		Linking IP3Rs to VDACs, fostering MAMs formation, and Ca^2+^ transfer	Szabadkai et al., 2006
VDACs	OMM porins		Interaction with GRP75 and IP3Rs, promoting Ca^2+^ uptake of mitochondria in MAMs	Maurya and Mahalakshmi, 2016
Tespa1	Regulator of Ca^2+^ flux in MAMs	Binding to GRP75	Maintaining MAMs integrity and affecting IP3R/GRP75/VDAC1 complex interactions	Matsuzaki et al., 2013
Sig1R	Chaperon at the ER	Forming complexes with BIP	Resulting in continuous mitochondrial Ca^2+^ influx via stabilizing IP3R at MAMs in case of separation from BIP by stimuli	Hayashi and Su, 2007
SERCA	Ca^2+^ uptake pump at the ER	Interacting with calnexin	Maintaining intracellular calcium homeostasis	Bollo et al., 2010
RyRs	Ca^2+^ uptake pump at the SR in cardiomyocytes	Forming RyR2-VDAC2 complex	Promoting the Ca^2+^ transfer of ERMCs in heart	Min et al., 2012
**(2) Lipid synthesis and transport proteins**				
ORP 5/8	PTPIP51 binding proteins in ER	Forming ORP5-PTPIP51; Competition with VAPB-PTPIP51	Mediating PS transfer; Expanding the ERMCs with PTPIP51	Pulli et al., 2018
ACAT	Regulate the balance of intracellular cholesterol	Synergizing with HMGCS and HMG-CoA reductase	Catalyzing the connection of long-chain fatty acids with free cholesterol to form cholesteryl esters	Li et al., 2021
DGAT	A major triacylglycerol biosynthetic enzyme	Interacting tightly with mitochondria	Catalyzing the final step of TG synthesis	Kornmann, 2020
PSS	Enzymes involved in PE biosynthesis	PSS-1 is highly enriched in MAM	Catalyzing PC and PE to generate PS	Vance and Steenbergen, 2005
StAR	OMM proteins	Binding to Tom22, VDAC2 and Sig1R	Mediating cholesterol transport; Undergoing steroidogenesis	Garay et al., 2017
**(3) MAM tethers proteins**				
VAPB	Tethers	Forming VAPB-PTPIP51; Suppressing ORP5-PTPIP51	Interacting with PTPIP51 to ensure the formation of MAMs; Regulating autophagy via Ca^2+^ transfer	Stoica et al., 2014
PTPIP51		Binding to VAPB and ORP5; Interaction with PTP1B	Ensuring the formation of MAMs as the linker complex; Modulating mitochondrial structure and function with ORP5/8	Gomez-Suaga et al., 2017a
VPS13A		Interacting with the ER protein VAPA	Promoting MAM formation and mitochondria stability; Involved in mitophagy	Yeshaw et al., 2019
EI-24		Forming a quaternary complex with IP3R/GRP75/VDAC1	Promoting MAM formation; Regulating calcium transfer; Cause apoptosis	Yuan L. et al., 2020
BAP31		Interacting with Tom40	Maintaining the integrity of ERMCs and regulating the mitochondrial respiration by BAP31-Tom40 complex	Namba, 2019
ERMES		Consisting of Mmm1, Mdm10, Mdm12, and Mdm34	Promoting phospholipid biosynthesis and calcium-signaling genes	Kornmann et al., 2009
**(4) MAM controlled proteins**				
PACS-2	MAM regulatory protein	Stabilizing BAP31	Required for MAM stabilization; Regulation of Ca^2+^ transfer and phospholipid synthesis; Inhibiting apoptosis	Moulis et al., 2019
Mfn2	GTPase located in ERMCs	Interacting with Mfn1/2 to form homo- or heterodimeric complexes	Promoting fusion of mitochondria and structural stabilization of MAMs; Facilitating Ca^2+^ transfer	Larrea et al., 2019
FUNDC1	Autophagy regulatory protein	Interacting with calnexin; Binding to IP3Rs	Promoting DRP1-dependent mitochondrial fission and mitophagy; Increasing the Ca^2+^ concentration in both the cytoplasm and the mitochondria	Wu et al., 2016
DsbA-L	Antioxidant enzyme	Forming DsbA-L/FATE1complex	Maintaining the integrity of MAMs; Reducing ER stress and apoptosis of ERMCs	Yang M. et al., 2019
FATE-1	Uncoupler of MAMs		Increasing the distance between mitochondria and ER and decreasing Ca^2+^ transfer of ERMCs	Doghman-Bouguerra et al., 2016

*ER, endoplasmic reticulum; ERMCs, ER-mitochondria contacts; MAM, mitochondria-associated ER membrane; OMM, outer mitochondrial membrane; IP3R, inositol 1,4,5-triphosphate receptor; GRP75, glucose-regulated protein 75 kDa in size; VDAC, voltage-dependent anion selective channel protein; Sig1R, Sigma-1 receptor; BIP, binding immunoglobulin protein; Tespa1, Thymocyte-expressed, positive selection-associated 1; SERCA, sarco/endoplasmic reticulum Ca^2+^-ATPase; RyRs, ryanodine receptors; ORP5/8, oxysterol-binding protein-related protein 5/8; PTPIP51, protein tyrosine phosphatase interacting protein 51; VAPB, Vesicle-associated membrane protein-associated protein B; BAP31, B-cell receptor-association protein 31; Tom40, translocase of the outer mitochondrial membrane 40; ERMES, The ER–mitochondria encounter structure; ACAT, acyl CoA-cholesterol acyl transferase; HMG-CoA, hydroxymethylglutaryl coenzyme A; HMGCS, HMG-CoA synthase; DGAT, diglyceride acyltransferase; TG, triglyceride; PSS, phosphatidylserine synthase; PS, phosphatidylserine; PE, phosphatidylethanolamine; PC, phosphatidylcholine; StAR, steroidogenic acute regulatory protein; Tom22, translocon of the outer membrane 22; VPS13A, Vacuolar protein sorting 13A; EI-24, etoposide-induced protein 2.4; PACS-2, phosphofurin acidic cluster sorting protein-2; BAP31, B-cell receptor-association protein 31; Mfn1/2, mitochondrial fusion protein 1/2; FUNDC1, FUN14 domain-containing 1; DRP1, dynamin-related protein 1; DsbA-L, disulphide-bond A oxidoreductase-like protein; FATE-1, fetal and adult testis-expressed 1.*

#### Ca^2+^ Regulatory Proteins

IP3Rs are highly expressed at MAMs. IP3Rs are spatially specific, and certain isoforms of IP3Rs, such as IP3R3, are specifically concentrated at MAMs. Earlier studies found that IP3Rs/GRP75/VDAC1 can form complexes in MAMs, and IP3Rs are expressed in the ER membrane (Bartok et al., 2019). VDAC1 is expressed in the OMM, and GRP75 connects IP3Rs with VDAC1, thereby connecting the ER membrane with the mitochondrial membrane to promote the formation of MAMs, and mediate ER-mitochondrial calcium transfer and affect mitochondrial function (Szabadkai et al., 2006). There is evidence that GRP75 deficiency leads to reduced Ca^2+^ exchange at ERMCs. Because disruption of the bridge perturbs Ca^2+^ flux into mitochondria but does not alter the close proximity of the ER and mitochondria, the IP3R/GRP75/VDAC1 protein complex is specialized for Ca^2+^ transfer at MAMs (Honrath et al., 2017). Thymocyte-expressed, positive selection-associated 1 (Tespa1) is expressed in MAMs and binds to GRP75, affecting IP3R/GRP75/VDAC1 complex interactions. Silencing Tespa1 expression reduces T cell receptor (TCR)-mediated mitochondrial and cytoplasmic calcium influx (Matsuzaki et al., 2013). Pyruvate dehydrogenase kinase 4 (PDK4) (Thoudam et al., 2019), glycogen synthase kinase-3β (GSK-3β) (Klec et al., 2019), and transglutaminase 2 (TG2) (D’Eletto et al., 2018) are also involved in the regulation of the IP3R/GRP75/VDAC complex, which affects MAM formation and ERMCs calcium transfer. We found that Nogo-B affects the binding of GRP75 and may regulate the formation of MAMs through the protein interactions of this complex (Yang Y. D. et al., 2019).

Sig1Rs are integral membrane proteins that possess two transmembrane domains and localize to the C-terminus of the ER. They are involved in multiple functions of brain tissue and other organs and play an important role in neuropsychiatric disorders, pain disorders, and tumor progression (Penke et al., 2018). Sig1Rs can form complexes with binding immunoglobulin protein (BIP) at MAMs, and after the ER receives a stimulating signal, Sig-1Rs separate from BIP and bind IP3Rs to stabilize their expression, which causes continuous mitochondrial calcium influx (Hayashi and Su, 2007). Other binding partners of Ca^2+^ such as calnexin and calreticulin are similarly expressed in MAMs and are involved in the regulation of ER-mitochondrial calcium signaling (Stahon et al., 2016).

RyR is a calcium release channel on ER/SRs. In mammals, there are three isoforms, namely skeletal muscle type (RyR1), cardiac muscle type (RyR2), and brain type (RyR3), which are encoded by the ryr1, ryr2, and ryr3 genes, respectively (Ogawa, 1994). It has previously been shown that RyRs are not only present on the ER/SRs, but also localized to the IMM and may be involved in Ca^2+^ uptake of mitochondria. The structural and functional properties of mitochondrial RyR (mRyR) are similar to those of RyR1 in skeletal muscles and different from those of RyR2 in cardiomyocytes, which were almost absent in the heart by immunoprecipitation and liquid chromatography-tandem mass spectrometry (LC-MS/MS) techniques (Beutner et al., 2005).

RyR2 is a 560 kDa molecule responsible for Ca^2+^ release from SR. RyR2 plays an important role in the Ca^2+^ at ERMCs in cardiomyocytes. Typically, VDAC1 physically interacts with IP3Rs via the GRP75 to mediate Ca^2+^ transport. In addition, RyR2 and VDAC2 are two key proteins for the functional ERMCs in cardiomyocytes, and RyR2-VDAC2 coupling may be indispensable. Previous study found that RyR2 was physically coupled to VDAC2 in the heart and co-localized with RyR2 in the subsarcolemmal region of cardiomyocytes (Min et al., 2012). In brief, RyR2-VDAC2 complex could also promote the uptake of mitochondrial Ca^2+^ and the function of ERMCs in cardiomyocytes.

#### Lipid Synthesis and Transport Proteins

Enzymes involved in various types of lipid metabolism are enriched in MAMs, such as phosphatidylserine synthase (PSS), phosphatidylserine decarboxylase (PSD), phosphatidylethanolamine N-methyl transferase (PEMT), long chain acyl-CoA synthase 4 (ACSL4/FACL4), acyl CoA-cholesterol acyl transferase (ACAT), lysophosphatidylinositol-acyltransferase-1 (LPIAT1), steroidogenic acute regulatory protein (StAR), diglyceride acyltransferase (DGAT), and glucose-6-phosphatase (G-6-Pase) (Kornmann, 2020).

Synthetases abundant on MAMs can locally synthesize phosphatidylserine (PS), phosphatidylethanolamine (PE), and phosphatidylcholine (PC), which are all major structural components of membranes. PSS-1 and PSS-2 are involved in PS biosynthesis in mammalian cells. Experiments revealed that PSS-1 was highly enriched in rat liver MAMs (Stone and Vance, 2000). PS transport is achieved through oxysterol-binding protein-related protein (ORP) 5 and ORP8, which are abundantly expressed in MAMs. These enzymes enriched in MAMs may support the direct transfer of lipids between the ER and mitochondria (Pulli et al., 2018). The PS generated by the ER is transferred to the mitochondria and converted to PE by a decarboxylation reaction. This process is the main source of mitochondrial PE, and mice lacking PSD activity develop abnormal mitochondrial function or even death during the embryonic period (Zhao and Wang, 2020). In addition to enzymes involved in phospholipid synthesis, the enzymes required for triacylglycerol, cholesterol, and glycosphingolipid synthesis are also enriched in MAMs, and thus, they are a core regulatory link in the balance of lipid metabolism (Vance, 2014).

#### Mitochondria-Associated Endoplasmic Reticulum Membranes Tethers Proteins

Vesicle-associated membrane protein-associated protein (VAP) B and protein tyrosine phosphatase interacting protein 51 (PTPIP51) are expressed in MAMs and interact to form linker complexes, ensure the formation of MAM structures, and mediate calcium transfer from ERMCs (Stoica et al., 2014). Knockdown of VAPB and PTPIP51 expression reduced calcium transfer levels by 86% (Gomez-Suaga et al., 2017a). VAPB and PTPIP51 are expressed at neuronal synapses, and after stimulation, VAPB-PTPIP51 interaction is enhanced and MAM formation is increased. Deficiency of VAPB and PTPIP51 reduced the number of dendritic spines and synaptic activity (Gomez-Suaga et al., 2019).

Vacuolar protein sorting (VPS) 13A is expressed in MAMs, and its FFAT domain interacts with the ER protein VAPA, which binds mitochondria via its C-terminus and anchors the ER and mitochondria (Park et al., 2016). After knockdown of VPS13A, MAM formation was reduced, mitochondria appeared fragmented, and mitophagy was inhibited (Yeshaw et al., 2019). The DNA damage response activates p53-mediated transcription of a variety of ER morphology proteins (Zheng et al., 2018). Among them, etoposide-induced protein 2.4 (EI24) forms an ER-mitochondrial linker with VDAC to promote MAM formation, mediate ER-mitochondrial calcium transfer, and cause apoptosis (Yuan L. et al., 2020).

B cell receptor–associated protein 31 (BAP31) is an integral ER membrane protein (Namba et al., 2013), which also plays a pivotal role in the constitution of the ERMCs by interacting with translocase of the outer mitochondrial membrane 40 (Tom40) to stimulate the translocation of ubiquinone oxidoreductase (mitochondrial complex I) core subunit 4 (NDUFS4), the component of complex I from the cytoplasm to the mitochondria. BAP31 is a key factor in maintaining the integrity of ERMCs with Tom40, and the BAP31-Tom40 complex is essential for activating mitochondrial respiration by stimulating NDUFS4 translocation in mitochondria by regulating the activity of the mitochondrial respiratory chain complex I. After loss of BAP31 function, it can affect the metabolism of cells, followed by a decrease in mitochondrial aerobic respiration-dependent ATP levels, triggering AMPK signaling and ultimately leading to autophagy (Namba, 2019).

The ER–mitochondria encounter structure (ERMES) is a complex which consists of mitochondrial morphology protein 1 (Mmm1), mitochondrial distribution and morphology protein 10 (Mdm10), Mdm12 and Mdm34 in yeast that reside in both the ER and the OMM except that Mdm12 is cytoplasmic. Mdm12, Mdm34, and Mmm1 possess a common SMP domain, which maintains ER contact with the mitochondrial membrane, promotes MAMs formation, and mediates ERMCs functions (Burgess et al., 1994). With the use of genome-wide mapping of genetic interactions, a study showed that the ERMES were functionally connected to phospholipid biosynthesis and calcium-signaling genes. In addition, phospholipid biosynthesis was impaired in mutant cells (Kornmann et al., 2009).

#### Mitochondria-Associated Endoplasmic Reticulum Membrane Controlled Proteins

##### Phosphofurin Acidic Cluster Sorting Protein-2

Phosphofurin acidic cluster sorting protein-2 was identified as the first MAM regulatory protein with a number of functions that can mediate ERMCs. Overall, PACS-2 regulates ER homeostasis and MAM formation (Simmen et al., 2005). Both PACS-2 and B-cell receptor-association protein 31 (BAP31) are located in the MAMs. Experiments have demonstrated that knockdown of PACS-2 resulted in BAP31-dependent mitochondrial fragmentation, deregulated synthesis of lipids, and translocation of BH3-interacting domain death agonists (Bid) to mitochondria, indicating that PACS-2 is required for MAM stabilization (Iwasawa et al., 2011). Mechanistically, PACS-2 depletion induces BAP31 cleavage to form P20. P20 triggers the release of Ca^2+^ from the ER to the mitochondria while regulating calcium homeostasis in the cell, which then recruits DRP1 to the mitochondria and subsequently induces mitochondrial fragmentation. Upon apoptotic stimulation, Bid translocates into the mitochondria via PACS-2 to initiate apoptosis (Simmen et al., 2005). Mammalian target of rapamycin (mTOR) complex 2 (mTORC2) located in MAMs phosphorylates PACS-2 by activating Akt to maintain the integrity of MAMs, indicating that phosphorylated PACS-2 is involved in the maintenance of the MAM structure. In addition, mTORC2-Akt-regulated phosphorylation at Ser437 of PACS-2 maintains the formation of MAMs (Betz et al., 2013). PACS-2 also regulates the transport of calnexin and transient receptor potential channel P2 (TRPP2) from MAMs to the plasma membrane (Kottgen et al., 2005). PACS-2 similarly mediates MAMs-regulated phospholipid synthesis (Hamasaki et al., 2013).

##### Mfn2

Mfn2 is a GTPase located on the OMM that stabilizes interactions between adjacent mitochondria and promotes fusion of mitochondria (de Brito and Scorrano, 2008). In recent years, it has been found that within the ER-mitochondrial membrane contact interface, Mfn2 localized in the ER interacts with Mfn2 or Mfn1 in mitochondria to form homo- or heterodimeric complexes and promote the structural stabilization of MAMs. Mutation of Mfn2 resulted in the dissociation of both membranes, morphological changes in the ER and mitochondria, and corresponding functional alterations in some patients but not all (Larrea et al., 2019).

A previous study found that ERMCs are approximately two times more abundant in Mfn2 KO cells than in wild-type (WT) cells examined by electron microscopy, which suggested that Mfn2 did not play a critical role in the ERMCs (Cosson et al., 2012). Not only the structural ERMCs but also the functions of them including Ca^2+^ transfer increased due to the Mfn2 reduction using a multiple study approaches (Filadi et al., 2015). Gp78/autocrine motility factor receptor (AMFR) is an E3 ubiquitin ligase in the ER that promotes MAM formation, where it can cause degradation of Mfn2 (Li L. et al., 2015). Based on these results, another perspective for ERMCs was proposed in which Mfn2 works as a tethering antagonist rather than a tether. In order to further determine the regulation of MAMs by Mfn2, it was experimentally found that MAM formation was reduced after knockdown of Mfn2, while the calcium transfer of ERMCs was inhibited, which demonstrated the positive regulation of MAM formation by Mfn2 (Zorzano et al., 2010). Other regulators such as mitochondrial ubiquitin ligase (MITOL) (Takeda et al., 2019) and trichoplein/mitostatin (TpMs), a keratin-binding protein, can also affect the integrity of MAMs and mitochondrial function by affecting the assembly and degradation of Mfn2 (Cerqua et al., 2010).

##### Disulphide-Bond A Oxidoreductase-Like Protein and Fetal and Adult Testis-Expressed 1

As previously reported, disulphide-bond A oxidoreductase-like protein (DsbA-L) and fetal and adult testis-expressed 1 (FATE-1) were expressed in MAMs. DsbA-L, an antioxidant enzyme, showed the function of reducing ER stress and maintained MAMs integrity while FATE-1 can regulate mitochondrial Ca^2+^ uptake and dissociate the MAMs (Doghman-Bouguerra et al., 2016; Tubbs et al., 2018; Yang M. et al., 2019). One study has showed that the MAMs was reduced in the kidneys of diabetic DsbA-L gene-deficient mice (DsbA-L^–/–^). Importantly, the overexpression of DsbA-L in HK-2 cells restored the high-glucose induced dysfunction of MAMs and reduced apoptosis. Interestingly, these beneficial effects were partially abolished by overexpression of FATE-1 (Yang M. et al., 2019). Previous studies implicated that FATE-1 is a cancer-testis antigen which has been identified as an uncoupler of MAMs (Doghman-Bouguerra et al., 2016; Tubbs et al., 2018). Under the control of steroidogenic factor-1 (SF-1), FATE1 upregulation increased the distance between mitochondria and ER and decreased mitochondrial Ca^2+^ uptake (Doghman-Bouguerra et al., 2016).

#### Lipids

After MAMs were treated with proteinase K, nearly 50% of their function remained (Kobuchi et al., 2012). Upon removal of cholesterol from membranes using methyl-β-cyclodextrin (MβC), the expression of Sig1Rs was reduced in the lipid raft regions of MAMs and would lead to calcium overload. Further experiments revealed that MβC inhibits PS lyases expressed in MAMs, the *de novo* synthesis function of PS is disturbed, and MAM formation is reduced (Fujimoto et al., 2012). The above results suggest that cholesterol may affect the function of ERMCs by altering the structure of biofilms at MAMs.

## The Function of Endoplasmic Reticulum-Mitochondria Contacts

**FIGURE 1:**
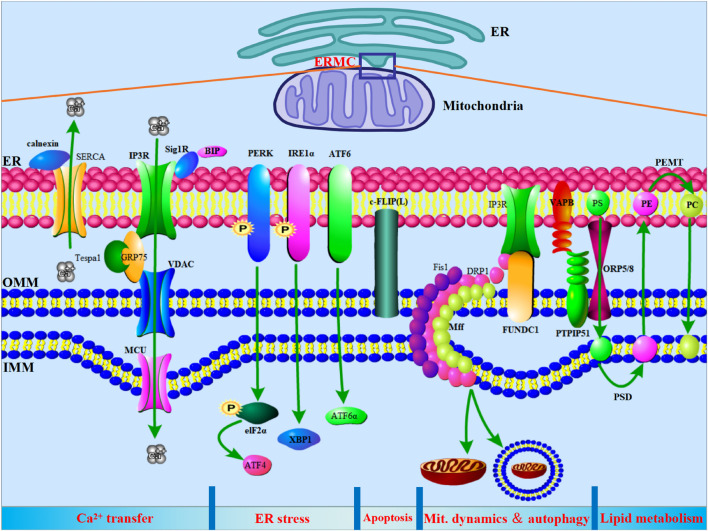
ER-mitochondria contacts (ERMCs) are involved in the regulation of Ca^2+^ transfer, ER stress, apoptosis, mitochondria dynamics, autophagy, and lipid metabolism. Top, a schematic diagram to show the ERMCs. Below, on a schematized segment of MAMs, the molecular contributors to the main known functions of ERMCs are summarized.

Studies have shown that core proteins and signaling pathways involved in various life activities of cells are expressed in ERMCs. In addition to earlier findings of calcium transfer and phospholipid metabolism, ERMCs are also involved in steroid synthesis, ER stress, mitochondrial dynamics, autophagy, and apoptosis (Figure 1).

### Endoplasmic Reticulum-Mitochondria Contacts and Calcium Transfer

Earlier studies revealed that mitochondrial calcium levels and the Ca^2+^ concentration of ERMCs significantly increased after HeLa cells were stimulated with IP3 (Lao and Chang, 2008). Subsequent studies confirmed that the IP3Rs/GRP75/VDAC1 protein complex is expressed in ERMCs and mediates the transfer of calcium (Moshkforoush et al., 2019). IP3Rs mainly regulate ER calcium efflux and exist in three isoforms. IP3R1 is widely distributed and particularly abundant in the brain, oocytes, and eggs. IP3R2 is expressed in the epithelium, myocardium, and skeletal muscle. However, IP3R3 is highly expressed in different cells and tissues. IP3R3 mainly mediates the release of ER calcium, while IP3R1 is mainly involved in the stabilization of cytosolic calcium (Bartok et al., 2019). Recently, it has been shown that Akt can phosphorylate IP3R3 at ERMCs and inhibit calcium release, thereby reducing the level of mitochondrial calcium and promoting cell proliferation (Marchi et al., 2008). mTORC2, promyelocytic leukemia protein (PML), and protein phosphatase 2A (PP2A) can activate Akt, promote its phosphorylation, and regulate calcium transfer in ERMCs, on the contrary, PTEN can counteract Akt activation and thus can inhibit the Akt- mediated phosphorylation of IP3R3 that protects from Ca^2+^ mediated apoptosis (Ando et al., 2018).

A variety of oxidoreductases are involved in the regulation of calcium transfer at ERMCs. Endoplasmic reticulum oxidoreductin 1α (Ero1α) is expressed in ERMCs and regulates calcium release from IP3Rs (Anelli et al., 2012). Endoplasmic reticulum resident protein (ERP) 44 belongs to the thioredoxin family and is localized in the ER lumen. ERP44 can directly bind IP3R1 and inhibit Ca^2+^ efflux, and this interaction is regulated by hydrogen ion concentration, Ca^2+^ concentration, and redox state (Higo et al., 2005). ER glutathione peroxidase 8 (GPX8) is expressed in ERMCs and can scavenge hydrogen peroxide generated by Ero1α mediation (Ramming et al., 2014).

Calnexin is associated with chaperones that bind newly synthesized glycoproteins and delay the process of protein folding. After detachment from the formed complex, the glycoprotein is transported to the Golgi when correctly folded. If misfolded, it is glycosylated again and enters into the calnexin cycle (Gutierrez et al., 2020). ERP57 in the protein disulfide isomerase (PDI) family is involved in this cycling process through binding to calnexin. Thus, calnexin prevents the accumulation of misfolded proteins within the ER (Lynes et al., 2013). At the ERMCs interface, calnexin interacts with sarco/endoplasmic reticulum Ca^2+^-ATPase (SERCA) 2b to maintain intracellular calcium homeostasis. Phosphorylation at S562 in the cytoplasmic domain of calnexin promotes binding to SERCA2b. This site is dephosphorylated during IP3-mediated ER calcium release, and the interaction of calnexin with SERCA2b is inhibited (Bollo et al., 2010). Alternatively, the phosphorylation status of calnexin and its interaction with PACS-2 also determine the distribution of calnexin protein in MAMs (Myhill et al., 2008).

### Endoplasmic Reticulum-Mitochondria Contacts and Lipid Metabolism

Lipids in the IMM and OMM cannot be synthesized in mitochondria. The close contact of the ER membrane with the mitochondrial membrane allows the rapid exchange of lipids between mitochondria and the ER. Studies have shown that enzymes involved in lipid metabolism are expressed in ERMCs and are involved in the metabolism of phospholipids, triglycerides, fatty acids, and steroids.

#### Phospholipid Metabolism

Earlier studies found that most of the mitochondrial PE is derived from the decarboxylation of PS after labeling serine or ethanolamine using [3H], and radiolabeled PS accumulates in ERMCs after administration of PS decarboxylation inhibitors (Steenbergen et al., 2005). PS was significantly increased in ERMCs after ATP deprivation. The above results indicate that PS can enter mitochondria through ERMCs after synthesis, and ATP is involved in this transport process (Vance and Steenbergen, 2005). Subsequent studies confirmed that PSS-1 and PSS-2 are expressed in ERMCs and catalyze PC and PE to generate PS. Subsequently, PS is transferred into the mitochondria, where it is decarboxylated by PSD1 at the IMM to generate PE. PE returns to the ER and methylates via PEMT2 at ERMCs to generate PC (Vance, 2015). It is the ERMCs that produce several major components that enable phospholipids to be efficiently synthesized and transported to the appropriate position (Vance, 1990; Stone and Vance, 2000; Voelker, 2000).

#### Steroid Synthesis

Recent studies have shown that ACAT1 is expressed in ERMCs. Intracellularly, ACAT1 is the only enzyme that catalyzes the connection of long-chain fatty acids with free cholesterol to form cholesteryl esters, which together with hydroxymethylglutaryl coenzyme A (HMG-CoA) synthase (HMGCS) and HMG-CoA reductase maintain and regulate the balance of intracellular cholesterol metabolism and play a key role in the development and progression of atherosclerosis (Rusinol et al., 1994; Li et al., 2021). The StAR protein transports cholesterol to undergo steroidogenesis, where it binds to VDAC2 at the OMM to mediate cholesterol trafficking to the OMM (Liu et al., 2006). Subsequently, cholesterol is catalyzed by cytochrome P450 side-chain cleavage (SCC) enzymes to generate pregnenolone. Pregnenolone returns to the ER to generate other classes of steroids. StAR is expressed in ERMCs and mediates cholesterol transport (Garay et al., 2017). In ERMCs, StAR binds the mitochondrial proteins Tom22 and VDAC2, and conversely, VDAC2 regulates the process of StAR entry into mitochondria. Upon knockdown of VDAC2, StAR is expressed but fails to bind ERMCs interface proteins in the mature protein form and enter the mitochondria, thereby inhibiting steroid synthesis (Prasad et al., 2015). In addition, StAR can bind to Sig1R and GRP78 before transferring cholesterol to mitochondria, which regulate the expression and activity of StAR. After knockdown of Sig1R, the synthesis of pregnenolone was decreased by 75–95% (Marriott et al., 2012). GRP78, a chaperone in the ER, is expressed in ERMCs and folds at the interface of ERMCs to activate StAR and promote its trafficking to the OMM. After knockdown of GRP78 expression, the expression and activity of StAR were significantly reduced (Prasad et al., 2017).

### Endoplasmic Reticulum-Mitochondria Contacts and Endoplasmic Reticulum Stress

The ER is involved in protein folding, lipid synthesis, and calcium storage. Under ER stress, misfolded proteins accumulate in the ER. Chaperones such as GRP78/BIP and three ER stress-sensing proteins, which are PKR-like ER kinase (PERK), activating transcription factor (ATF) 6, and inositol-requiring enzyme 1α (IRE1α), initiate unfolded protein response (UPR) after activation of recognition to repair misfolded proteins and maintain proteostasis (Oakes and Papa, 2015). Appropriate UPR levels can promote cell survival, whereas when ER stress is sustained or excessive, the ER apoptotic pathway is activated and promotes apoptosis.

#### The PKR-Like Endoplasmic Reticulum Kinase- Mediated Signaling Pathway

PKR-like endoplasmic reticulum kinase is a type I transmembrane protein, and its cytoplasmic fraction contains a serine/threonine kinase domain (Wang et al., 2020a). Under physiological conditions, heat shock protein (HSP) 90 and BIP bind the cytoplasmic and ER domains of PERK and stabilize PERK. Under ER stress, BIP binds misfolded proteins, thereby releasing PERK and causing PERK dimerization and autophosphorylation activation. Subsequently, PERK activates eukaryotic initiation factor 2 (eIF2) to inhibit translation initiation. PERK phosphorylation ensures cell survival, and results in differentiation and metabolism-related substances, such as nuclear factor E2-related factor 2 (Nrf2), forkhead box O (FOXO), and diacylglycerol (DAG) (Xu Y. et al., 2020). In addition to inhibiting total mRNA translation, PERK activates the transcription of some genes, including ATF4, ATF5, and amino acid transport carriers. ATF4 enters the nucleus to activate the transcription of genes related to antioxidant response and amino acid synthesis and transport and promote cell survival. However, ATF4 can also activate the expression of pro-apoptotic transcription factor C/EBP homologous protein (CHOP) and mediate the ER stress apoptotic pathway (Verfaillie et al., 2012). Recent studies have found that PERK is expressed in ERMCs and rapidly transfers lipid peroxides from the ER into mitochondria, causing oxidation of cardiolipin in mitochondria and release of cytochrome *c*, and then promotes apoptosis (Rozpedek et al., 2016). In addition, the PERK-eIF2α-ATF4 pathway activates the expression of SERCA1 in ERMCs, which can promote ERMCs formation and feedback enhancement of the PERK-eIF2α-ATF4 pathway. Knockdown of SERCA1 expression can inhibit ER stress-induced mitochondrial calcium overload and apoptosis (Guo et al., 2016).

#### The ATF6-Mediated Signaling Pathway

ATF6 is a type II transmembrane protein containing the cytoplasmic cAMP response binding protein domain. Under ER stress, ATF6 is released from the ATF6/BIP protein complex and further processed by proteases site-1/2 protease (S1P/S2P) in the Golgi apparatus to produce activity. The cleaved ATF6α activates adaptive responses, whereas ATF6β may inhibit ATF6α function (Huang et al., 2018). During ER stress, ATF6 expression increases, which can promote the formation of ERMCs, increase ER-mitochondrial calcium transfer, and enhance mitochondrial respiration, thereby inhibiting cell proliferation and promoting apoptosis (Burkewitz et al., 2020).

#### The Inositol-Requiring Enzyme 1α-Mediated Signaling Pathway

Inositol-requiring enzyme 1α is an endonuclease in the ER that is widely distributed and binds HSP72/HSP90/BIP under physiological conditions (Shemorry et al., 2019). ER stress can cause IRE1α oligomerization and phosphorylation, activating its kinase and endoribonuclease activities. Subsequently, IRE1α activates cleavage to generate X-box binding protein 1 (XBP1), which enters the nucleus to promote gene transcription for protein folding, trafficking, and degradation of ER-associated proteins (Zhang et al., 2018). Recently, it was shown that IRE1α is expressed in ERMCs, and MITOL can mediate K63 chain ubiquitination at Lys481 of IRE1α, thereby inhibiting the polymerization reaction of IRE1α. Under ER stress, reduced expression of MITOL can increase the endonuclease activity of IRE1α and promote apoptosis development (Takeda et al., 2019).

### Endoplasmic Reticulum-Mitochondria Contacts and Mitochondrial Dynamics

Mitochondria are highly motile organelles in cells that continuously fuse and divide. The fusion of two adjacent mitochondria causes the redistribution of mitochondrial DNA and protein, forming an evenly distributed mitochondrial network (Chen et al., 2003). Mitochondrial fission and fusion are precisely regulated by calcium and calcium-dependent kinases, phosphatases, metabolism, and intracellular oxidative status. A series of proteins is involved in the regulation of mitochondrial dynamics, such as Mfn1, Mfn2, and OPA1, as well as mitochondrial fission protein 1 (Fis1) and DRP1. Mfn1 mediates fusion of the OMM with Mfn2, whereas OPA1 integrates the inner mitochondrial membrane (Tilokani et al., 2018). Upon phosphorylation of Mfn1 by extracellular regulated protein kinase (ERK), mitochondria appear fragmented and BAK oligomerizes, which increases mitochondrial membrane permeability and initiates apoptosis (Pyakurel et al., 2015). Cyclin B1/cyclin-dependent kinase (CDK) 1 activates DRP-1 in response to growth factors and promotes DRP-1 phosphorylation to initiate the intracellular transfer of DRP1. DRP1 is located in the cytoplasm in the inactive state and translocates to the OMM upon activation. Subsequently, DRP1 assembles to form multimers that bind and divide mitochondria, and DRP1 phosphorylation inhibits mitochondrial fission (Kizaki et al., 1988).

The initiating role of ERMCs on mitochondrial fission is more evident. The ERMCs sites determine the location of the bound and divided mitochondria (Friedman et al., 2011). DRP1 assembles and recruits other split proteins Fis1, mitochondrial fission factor (Mff), and mitochondrial dynamics proteins of 49 and 51 kDa (MiD49/MiD51) at the ERMCs to form mitochondrial split protein polymers, which tighten mitochondria and initiate fission (Ji et al., 2017). In a real-time observational study, 84% of mitochondrial fission events occurred at sites of ERMCs. During mitochondrial segmentation, ERMCs points persist. After mitochondrial fission, the progeny mitochondria move distally, and the other maintains tight junctions with the ERMC points and remains relatively quiescent. Subsequently, the mitochondrial membrane elongates at the site of fission to form a tubular intermediate form until mitochondrial fission is complete (Guo et al., 2018). The above results indicate that the ERMCs not only mark the mitochondrial fission site but also act to stabilize mitochondria during mitochondrial fission. In addition, ERMCs proteins can regulate the activation of DRP1. The activity of DRP1 is reduced after phosphorylation of DRP1 by PKA, which expands the ER lumen, promotes the elongation of mitochondria, and increases ERMCs formation (Bravo-Sagua et al., 2019).

In addition to its involvement in mitochondrial fission, it was recently reported that ERMCs are similarly involved in mitochondrial fusion (Basso et al., 2018). Fifty-nine percent of mitochondrial fusion events occur at the ERMCs, with the ER being between two adjacent mitochondria. It was found that without the involvement of the ER, mitochondrial membrane fusion is a lengthier process as compared to having the involvement of the ERMCs (Guo et al., 2018). The results showed that ERMCs can accelerate the process of mitochondrial fusion, although the molecular mechanism requires further elucidation. ERMCs are similarly involved in mitochondrial DNA replication and predate mitochondrial fission (Qin et al., 2020).

### Endoplasmic Reticulum-Mitochondria Contacts and Autophagy

Autophagy is a process whereby intracellular components are degraded and recycled (Mizushima and Komatsu, 2011). After wrapping damaged organelles or proteins, lysosomes degrade their contents. The unc-51-like autophagy activating kinase (ULK) 1/2 complex initiates and activates the VPS34 complex. Subsequently, LC3-II which produced from cleavage of microtubule-associated protein light chain 3 (LC3) by ATG family proteins mediates the extension of isolated membranes (Parzych and Klionsky, 2014). Isolated membranes encapsulate damaged organelles, and cytoplasmic components continuously extend to produce phagocytic vacuoles, which form autophagosomes after blocking. In addition, p62 protein can bind LC3, enter autophagosomes, and undergo degradation by fused lysosomes. It was observed that total intracellular p62 protein levels were inversely correlated with autophagic flux (Seibenhener et al., 2013).

With adequate nutrition, ATG14 complexes are diffusely distributed in the ER membrane. In starvation-induced autophagy, ATG14 is specifically recruited in ERMCs by the ER-resident SNARE proteinsyntaxin17 (STX17) (Hamasaki et al., 2013). Other early autophagy marker proteins such as beclin-1 and zinc finger FYVE domain-containing protein 1 (ZFYVE1)/double FYVE-containing protein 1 (DFCP1) are also similarly expressed at ERMCs (Hamasaki et al., 2013). Knocking down ERMCs linkers such as PACS-2 or Mfn2 can inhibit the expression of autophagic proteins in ERMCs and prevent autophagy initiation (Hamasaki et al., 2013; Hu et al., 2021). In addition to autophagic proteins expressed in ERMCs, ganglioside (GD3) is similarly involved in the initiation of autophagy (Matarrese et al., 2014). GD3 interacts with the cytoskeletal network and can be rapidly transferred from the cell membrane to various components within the cell, including ERMCs. GD3 not only binds the conventional class III phosphatidylinositol-3-kinase (PI3K) complex to initiate autophagy, but also can interact with LC3 after autophagy initiation. Inhibition of ceramide synthase and GD3 synthase significantly inhibited the formation of autophagosomes. Upon autophagy induction, calnexin expression increased in ERMCs and promoted autophagy (Garofalo et al., 2016).

Mitophagy can selectively remove damaged mitochondria, improve mitochondrial energy metabolism disorders, and prevent the release of pro-apoptotic proteins in mitochondria, which is essential for the quality maintenance of mitochondria (Bravo-San Pedro et al., 2017). After mitochondrial damage, PTEN induced putative kinase 1 (PINK1) can accumulate on the membrane surface of damaged mitochondria as well as recruit and phosphorylate ubiquitin and parkin RBR E3 ubiquitin protein ligase (PARK2), which mediate ubiquitination labeling of OMM proteins and proteasomal degradation, and inhibit the fusion of damaged mitochondria (Tang et al., 2018). Subsequently, these organelles form autophagosomes by activation of specific ubiquitin-binding receptor proteins such as sequestosome 1 (SQSTM1)/p62, which fuse with lysosomes to complete mitophagy (Yamada et al., 2019). Upon stimulation with the mitophagy agonist carbonyl cyanide 3-chlorophenylhydrazone (CCCP), PINK1 and beclin-1 are expressed in ERMCs, and promote the formation of omegasomes produced by ERMCs. At the same time, PARK2 expression at the ERMCs increased (Gelmetti et al., 2017). However, knockdown of PINK1 expression mediated reduced beclin-1 expression in ERMCs through the non-PARK2 pathway (Erpapazoglou and Corti, 2015). FUN14 domain-containing 1 (FUNDC1) is expressed in ERMCs and interacts with calnexin. Upon stimulation, FUNDC1 detaches from the calnexin complex and recruits dynamin-1-like protein (DNM1L)/DRP1 to initiate mitochondrial fission. Knockdown of FUNDC1, DNM1L, and CANX increased mitochondrial length, inhibited the binding of autophagosomes to mitochondria, and inhibited mitophagy under hypoxia (Wu et al., 2016).

### Endoplasmic Reticulum-Mitochondria Contacts and Apoptosis

Regulation of calcium levels in ERMCs plays a pivotal role in apoptosis. Numerous proteins are involved in the regulation of the calcium transfer axis in ERMCs, thereby precisely regulating cell survival and death (Suresh, 2019). The long form of c-FLIP (c-FLIP_L_) is an isoform of cellular FLICE-like inhibitory protein (c-FLIP) that is expressed in the ERMCs. Knockdown of c-FLIP_L_ caused ER fragmentation and expansion of peripheral tubular ERs, and inhibited the formation of ERMCs (Jeong et al., 2015). Further studies showed that c-FLIP_L_ knockdown suppressed reticulon 4 (RTN-4) expression, and c-FLIP_L_ can inhibit apoptosis by inhibiting calcium transfer in ERMCs (Marini et al., 2015). Bcl-xl belongs to the Bcl-2 family of proteins, which are expressed in ERMCs and bind to each other with IP3R3. The overexpression of Bcl-xl promotes calcium transfer in ERMCs, decreases the NAD/NADH ratio, and enhances the oxidase activity of the electron transport chain (Bessou et al., 2020). BCL2-like 10 (Bcl2l10) binds to the IP3R and inhibits calcium release from the ER. IP3R binding protein released with IP3 (IRBIT) requires regulation of the IP3R through Bcl-2l10 binding. Cell fraction separation revealed that both Bcl2l10 and IRBIT are expressed in ERMCs, regulate the formation of ERMCs, and synergistically inhibit IP3R-mediated calcium release (Bonneau et al., 2016).

Studies have shown that cell necrosis is similarly regulated and is known as necroptosis. Necroptosis is involved in host defensive responses, chronic inflammation, and tissue damage, such as inflammation, hemolysis, and atherosclerosis (Frank and Vince, 2019). The formation of receptor interacting protein kinase-1 and -3/mixed lineage kinase domain-like protein (RIP1/RIP3/MLKL)-containing complexes in necrotic bodies can initiate the necroptosis pathway (Zhou et al., 2021). RPI1/RIP3/MLKL is expressed in ERMCs, and its content is higher than that in mitochondria. The process is mediated by the interaction of RIP3 and MLKL (Chen et al., 2013). The above results suggest that ERMCs may be the initiation site for accumulation of necrosomes and initiation of necroptosis, and further studies are needed to investigate the mechanism involved.

In conclusion, a growing body of studies has shown that core proteins involved in cellular biological processes are enriched in ERMCs, thereby exerting biological functions. Therefore, in pathological conditions, ERMCs can also participate in the development and progression of diseases.

## The Critical Role of Endoplasmic Reticulum-Mitochondria Contacts in Remodeling and Cardiovascular Remodeling-Associated Diseases

### Endoplasmic Reticulum-Mitochondria Contacts in Remodeling of the Heart

#### Endoplasmic Reticulum-Mitochondria Contacts and the Pathophysiology of Cardiac Remodeling

Myocardial remodeling refers to the pathological changes such as myocardial hypertrophy, myocardial apoptosis, and interstitial fibrosis induced by various pathological stimuli such as inflammation, oxidative stress, ischemia-reperfusion, and mechanical tension in the heart (Huang et al., 2020). Additionally, progressive damage to mitochondrial function, imbalance of energy homeostasis, and alteration of cardiac metabolism are important characteristics of cardiac remodeling (Sekaran et al., 2017; Ding et al., 2020).

**FIGURE 2:**
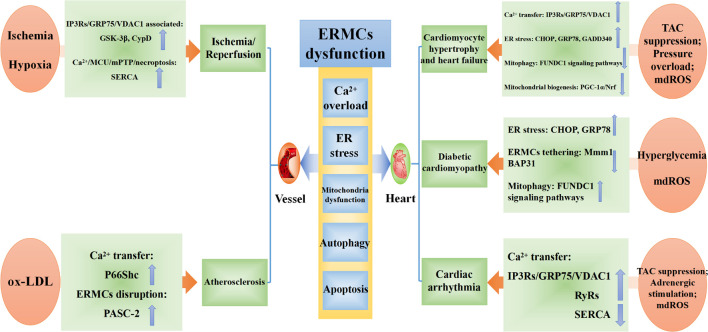
The pathophysiology and underlying mechanisms of ERMCs dysfunction in pathogenesis of cardiovascular remodeling.

The complex network formed by mitochondrial remodeling and the calcium buffer maintained by the ER and mitochondria are the premise for the maintenance of cardiomyocyte physiological function (Eisner et al., 2017). Based on the particularity of cardiomyocytes, there are essential differences in ERMCs compared with other types of cells, and they are especially prominent in the regulation of calcium homeostasis and calcium buffering involved in the ER and mitochondria (Chaanine et al., 2020). Calcium is a key second messenger regulating mitochondrial redox and energy metabolism, and during cardiomyocyte contraction, spontaneous Ca^2+^ oscillations evoked by the endoplasmic reticulum and cytoplasmic Ca^2+^ spikes propagate to the mitochondrial matrix to stimulate oxidative energy production (Jouaville et al., 1999). It has also been suggested that Ca^2+^ release via the IP3R-GRP75-VDAC1 complex is necessary to maintain the bioenergy of cardiomyocytes (Cárdenas et al., 2010). A massive release of ER Ca^2^^+^ into the cytoplasm causes mitochondrial Ca^2^^+^ overload as well as persistent opening of the mitochondrial permeability transition pore (mPTP), which can activate the mitochondrial apoptotic pathway and lead to cardiomyocyte apoptosis (Eisner et al., 2013). Moreover, continuous studies have confirmed that MAM-associated proteins are widely involved in multiple links of cardiovascular remodeling, and ERMCs is an important mechanism in the pathogenesis of heart diseases such as heart failure, myocardial hypertrophy, arrhythmia, and cardiomyopathy (Lopez-Crisosto et al., 2017; Silva-Palacios et al., 2020) (Figure 2).

#### Endoplasmic Reticulum-Mitochondria Contacts in Cardiomyocyte Hypertrophy and Heart Failure

Cardiac hypertrophy is a common type of remodeling that occurs in the heart under physiological and pathological overload. In sustained pathological overload, cardiomyocytes and the heart undergo enlargement that is accompanied by a decrease in the ventricular wall as well as interventricular septal stress and cardiac remodeling, leading to heart failure (Oka et al., 2014; Nakamura and Sadoshima, 2018). Studies have shown that the topology of mitochondria and the ER in cardiomyocytes is the center of signal transduction and calcium homeostasis, and crosstalk and alterations between the SR/ER and mitochondria have been involved in the pathogenesis of myocardial hypertrophy and heart failure (Reddish et al., 2017; Wacquier et al., 2019).

Overactivation of endoplasmic reticulum stress (ERS) and perturbation of mitochondrial function play a significant role in compensatory cardiac hypertrophy and progression to overt systolic heart failure (Chaanine et al., 2013; Ma et al., 2021). Intraperitoneal injection of tunicamycin (TN) is an interesting model that can be used to study the functional effects of cardiac ER stress. Prola et al. (2019) evaluated the exact physiological effect of TN-induced ER stress in mice, and the study found that TN injection resulted in a reduction of cardiac contractility and ultrastructural cytoarchitectural alterations in cardiomyocytes after 72 h. The expression of ER stress markers GRP78, CHOP, and growth arrest and DNA damage-inducible protein 34 (GADD34) increased, while the expression of peroxisome proliferator-activated receptor-gamma co-activator-1α (PGC-1α), a major regulator of mitochondrial biogenesis, and its downstream target Nrf1 were significantly inhibited. Mitochondria showed metabolic remodeling, slowed oxidative rate, and impaired Ca^2+^ uptake (Prola et al., 2019). These results provide evidence that the ER stress induced significant impairment of cardiac energy metabolism and function. In addition, SR as a modified ER, and mitochondrial-SR calcium crosstalk and cardiomyocyte Ca^2+^ transfer are closely linked to the development of heart failure (Eisner et al., 2013). The efficiency of Ca^2+^ transfer is affected by the distance between the ER and mitochondria, and in norepinephrine (NE)-induced hypertrophic cardiomyocytes, IP3R is dysfunctional. Mitochondrial Ca^2+^ uptake capacity is generally decreased, the average distance between the ER and mitochondria is increased, and ERMCs are decreased (Gutierrez et al., 2014).

Recently, MAM dysfunction was also implicated in the onset and progression of heart failure (HF) (Luan et al., 2021). Disruption of ERMCs has been found in Sig-1R, Mfn1/2, and FUNDC1-related HF (Silva-Palacios et al., 2020). The studies focused on the involvement of these proteins in HF pathogenesis are summarized below:

Duchenne muscular dystrophy (DMD) is a fatal disease featuring progressive cardiomyopathy, and compelling evidence supports the central role of mitochondrial dysfunction and impaired Ca^2+^ homeostasis in the pathogenesis of DMD (Meyers and Townsend, 2019). In dystrophin-deficient mice (mdx mice), IP3R1-VDAC1 interaction as well as GRP75-VDAC1 ligation were significantly enhanced, IP3R1 and its regulatory subunit Sig-1R were significantly increased, and the mitochondrial Ca^2+^ content was also significantly increased. It was shown that in mdx cardiomyocytes, IP3R1 and VDAC enhanced the physical interconnection of SR/ER-mitochondria. Additionally, increased IP3R1-GRP75-VDAC1 interaction is thought to enhance the direct channel of Ca^2+^ from SR/ER to the mitochondria (Angebault et al., 2020).

Mfn1/2-related proteins and VDAC2 probably have a role in stabilizing SR–mitochondrial interactions in cardiac muscle. Mfn1/2 is the protein most likely to be involved in tethering between SR and cardiac mitochondria (Chen et al., 2012; Hall et al., 2016). In the hearts of Mfn1and Mfn2 cardiac knockout mice, reduced mitochondrial size and contact length of ERMCs, as well as severe cardiomyopathy and impaired cardiac function, were observed (Papanicolaou et al., 2011, 2012a,b).

The OMM protein FUNDC1 mediates the formation of MAMs (Wang C. et al., 2021). One study has shown that interruption of FUNDC1 and IPR2 interaction reduces the level of Ca^2+^ in the mitochondria and cytoplasm, triggering abnormal mitochondrial fission and cardiac dysfunction. In heart tissue from patients with heart failure, it has also been demonstrated that the FUNDC1/MAMs/CREB/Fis1 signaling axis is significantly inhibited. FUNDC1 can degrade IP3R3 through FBXL2 to maintain Ca^2+^ homeostasis and mitochondrial function, and FUNDC1 knockout exacerbates high-fat diet-induced IP3R3 elevation, calcium overload, mitochondrial dysfunction, and cardiac remodeling in FUNDC1-KO mice (Ren et al., 2020).

Overall, ERMCs seem to have a strong role in cardiomyocyte hypertrophy and heart failure. First, mitochondrial dysfunction induced by excessive ER stress can affect myocardial energy metabolism and aggravate HF. In addition, the structure and dysfunction of MAMs overlap with the pathophysiological mechanism of HF. In particular, MAM-related proteins such as IP3R1, VDAC1, Mfn1/2, and FUNDC1 are directly involved in the pathophysiological process of cardiac remodeling. In summary, inhibiting the over-activation of ERS and the disturbance of mitochondrial function, decreasing the disorder of SR/ER-mitochondrial Ca^2+^ crosstalk, and correcting the abnormal expression of key proteins of MAMs will contribute to the prevention and treatment of pathological myocardial remodeling. Therefore, ERMCs are considered to be a key factor in the onset of cardiac hypertrophy and heart failure, and a potential therapeutic target.

#### Endoplasmic Reticulum-Mitochondria Contacts in Diabetic Cardiomyopathy

At present, the pathogenesis of DCM remains unclear, and studies have shown that it is mainly related to glucose metabolism disorders, lipotoxicity, myocardial fibrosis, oxidative stress, and insulin resistance (Liu et al., 2015; Tate et al., 2017; Zhao et al., 2019; Luo et al., 2020; Xiong et al., 2020). In recent years, studies have found that ER stress and mitochondrial apoptosis pathways may be involved in the progression of DCM, and myocardial cells in DCM patients are accompanied by glucose and lipid metabolism disorders, producing large amounts of reactive oxygen species (ROS). This leads to ER swelling and mitochondrial structure and function disorders, which in turn cause myocardial hypertrophy, interstitial fibrosis, myocardial necrosis, and apoptosis (Zhou et al., 2018b; Ljubkovic et al., 2019; Zhang J. et al., 2021).

In addition, MAMs’ constitutive proteins may be involved in the pathogenesis of diabetic cardiomyopathy, for example, Mfn2 and FUNDC1. These protein bridges of MAMs could be potential therapeutic targets for DCM in the future (Wu et al., 2019; Zhang J. et al., 2021). A recent work showed that Mfn2 promotes ERMCs in cardiomyocytes subjected to hyperglycemic conditions. Mfn2 siRNA significantly attenuated mitochondrial Ca^2+^ overload, increased mitochondrial membrane potential, and decreased ER swelling-induced cardiomyocyte death. Transmission electron microscopy micrographs suggested that Mfn2 silencing markedly increased the mean distance between the ER and the OMM (Yuan M. et al., 2020). Additionally, Mfn2 deficiency decreased the production of mitochondrial ROS and the frequency of mitochondria-dependent apoptosis in cardiomyocytes (Zhang J. et al., 2021). Therefore, the regulation of Mfn2-related MAMs may represent a potential therapeutic pathway for the treatment of DCM.

It has been observed that disruption of MAM integrity affects insulin signaling *in vivo* and *in vitro*, and ER-mitochondrial miscommunication was found to be an early event that induced insulin resistance before mitochondrial dysfunction occurred in type 2 diabetes (T2D) mice (Tubbs et al., 2018). For example, FUNDC1 is a highly conserved outer-membrane protein in mitochondria, and mediates the formation of MAMs (Zhang, 2021). Wu et al. (2019) observed that the level of FUNDC1 was increased in cardiac tissue from diabetic patients and Type 1 diabetic mice model. FUNDC1 overexpression promoted MAM formation, mitochondrial Ca^2+^ increase, and mitochondrial dysfunction (Wu et al., 2019). Moreover, cardiomyocyte-specific FUNDC1 knockdown eliminated diabetes-induced MAM formation, resulting in reduction of mitochondrial Ca^2+^ overload, mitochondrial fragmentation, and apoptosis, and increased mitochondrial functional capacity and cardiac function (Wu et al., 2019). This evidence indicated that FUNDC1 may serve an important role in diabetes-induced cardiac MAM formation. In addition, cardiac-specific FUNDC1 suppression prevented streptozotocin-induced cardiac abnormalities in diabetic mice and protected the function of the heart (Wu et al., 2019). It can be speculated that hyperglycemia-driven FUNDC1-related MAM formation was implicated in the mitochondrial Ca^2+^ overload and mitochondrial dysfunction. Interestingly, the reticulum–mitochondria Ca^2+^ miscoupling was found to disrupt the mitochondrial bioenergetics and cardiomyocyte contraction in an obesogenic T2D mouse model (Dia et al., 2020).

These findings suggest that the interaction between the ER and mitochondria via MAMs is hindered in the initiation and development of DCM, and this promotes Ca^2+^ overload, oxidative stress, mitochondrial dysfunction, and apoptosis, which eventually leads to myocardial remodeling and cardiac dysfunction.

#### Endoplasmic Reticulum-Mitochondria Contacts in Cardiac Arrhythmia

The mechanism of arrhythmia (including atrial arrhythmia) includes trigger activity and reentry, of which trigger activity is closely related to calcium cycle disorder and abnormalities of Ca^2+^ homeostasis (Sipido, 2006; Peyronnet et al., 2016). Ca^2^^+^ is a signaling ion in the heart, and is one of the most prevalent signal transduction molecules. It mediates a diverse array of biological functions including mitochondrial energy production, cardiomyocyte excitation-contraction (ECC), and triggering of programmed cellular death (Landstrom et al., 2017). Recent studies demonstrate that in the fast-paced HL-1 atrial muscle cell model for atrial tachycardia remodeling, ER stress induces activation of the MAPK pathway through mitochondrial-related apoptosis, which may be a mechanism involved in the occurrence of atrial fibrillation (Shi et al., 2015).

The ER and mitochondria act as intracellular calcium stores involved in Ca^2+^ storage and flow. RyR, IP3R, and SERCA2a are major channel proteins for Ca^2+^ release and uptake on the SR (Connell et al., 2020; Zhihao et al., 2020). In addition, IP3R1 and its chaperone protein GRP75, and the calcium transport channel VDAC1 of the OMM comprise the IP3R1-GRP75-VDAC1 complex involved in regulating ER-mitochondrial calcium flow (Pedriali et al., 2017). The presence of specialized proteins that connect the SR to the mitochondria ensures local calcium flux between these organelles. Recent evidence suggests that acquired modifications of ER-mitochondrial Ca^2+^ -handling proteins cause abnormal calcium flow between organelles, and their resulting disturbed calcium balance is associated with arrhythmias (Salazar-Ramirez et al., 2020). The altered SR Ca^2+^ handling proteins, such as RyRs and IP3R, play an important role in maintaining appropriate cardiomyocyte excitability, which may be initiated and intensified when mitochondria are dysfunctional (Salazar-Ramirez et al., 2020). Moreover, the junctional sarcoplasmic reticulum (jSR) is an important and specialized ER subdomain that concentrates resident proteins to regulate Ca^2+^ release in adult cardiomyocytes (Sleiman et al., 2015), jSR, mitochondria, and transverse-tubules (TTs), which are specialized sarcolemma invaginations, are tightly regulated and form an important and highly repetitive functional structure along the cell (De la Fuente and Sheu, 2019). Mitochondrial Ca-targeted fluorescent probes have revealed that mitochondria Ca^2+^ transients are synchronized with SR Ca^2+^ fluxes (Salazar-Ramirez et al., 2020). Different research groups provided evidence indicating that erratic abnormal action potential (AP) generation relies on pathological mitochondria-derived ROS (mdROS), and interaction between mitochondria and the SR (Salazar-Ramirez et al., 2020). Li Q. et al. (2015) developed a multiscale guinea pig cardiomyocyte model that included the mitochondria-SR microdomain for the first time. Simulations showed that mdROS bursts increased the cytosolic Ca^2+^ by inhibiting SERCA and stimulating RyRs, which elicited Ca^2+^ overload and abnormal AP (Li Q. et al., 2015). Therefore, it is important to evaluate the specific role of the interaction between mitochondria and the SR in Ca^2+^ overload-mediated cardiac arrhythmogenesis. This underscores the importance of considering SR-mitochondrial targets in designing new antiarrhythmic therapies.

### Endoplasmic Reticulum-Mitochondria Contacts in the Remodeling of the Vasculature

#### Endoplasmic Reticulum-Mitochondria Contacts and the Pathophysiology of Vasculature Remodeling

Vascular remodeling refers to the adaptive changes in structure and function that occur in response to various physiological and pathophysiological changes closely related to aging and vascular diseases (Ma et al., 2020). The concept of vascular remodeling was first proposed by Baumbach and Heistad (1992), and vascular remodeling is not only an important pathological basis for the progression of related diseases such as hypertension and atherosclerosis, but it is also the cause of the development of such diseases (Cai et al., 2021). Analyzing the process of vasculature remodeling at the cellular level is, therefore, of vital importance to more thoroughly understand the underlying mechanisms that lead to its development and elucidate new potential therapeutic targets to prevent it. Vascular remodeling is the production of a variety of cellular and molecular pathways, and the vascular wall is mainly composed of endothelial cells, smooth muscle cells, extracellular matrix, and adventitial fibroblasts (Méndez-Barbero et al., 2021). Functional changes in endothelial cells, and changes in proliferation, hypertrophy, and apoptosis of vascular smooth muscle cells as well as changes in adventitia and extracellular matrix are the cytological basis of vascular remodeling (Tanaka and Laurindo, 2017). More and more studies suggested an association between ERMCs and cardiovascular remodeling pathogenesis, thus implicating the participation of the SR/ER–mitochondria axis (Gomez et al., 2016; Garrido-Moreno et al., 2019; Yuan M. et al., 2020). Endoplasmic reticulum stress, Ca^2+^ homeostasis imbalance, and mitochondrial dysfunction are involved in vascular cell proliferation and apoptosis by regulating redox balance, intracellular ion homeostasis, and cellular metabolism (Lebiedzinska et al., 2009). A recent study demonstrated that Mfn2 overexpression causes mitochondrial fusion into tubular networks and attachment to the ER, which in turn prevents proliferation of vascular smooth muscle cells (VSMCs) (Li D. et al., 2015). A 2015 study found that ERMCs plays a critical role in the endothelium during reperfusion injury, and may be a new molecular target for endothelial protection. In this context, acetylcholine attenuates both intracellular and mitochondrial Ca^2+^ overload and protects endothelial cells from hypoxia/reoxygenation (H/R) injury, through disrupting the ER-mitochondria interaction. Importantly, inhibition of VDAC1 or Mfn2 reduced mitochondrial Ca^2+^ overload and endothelial cell death after H/R (He et al., 2015). Therefore, it is very important to understand the regulatory mechanism and targeted proteins involved in maintaining vascular remodeling related to ERMCs in order to explore new targets for the prevention or treatment of cardiovascular diseases.

#### Endoplasmic Reticulum-Mitochondria Contacts in Ischemia/Reperfusion

Myocardial ischemia/reperfusion (I/R) injury is defined as a pathophysiological phenomenon in which the structural damage and dysfunction of cells or tissues are instead progressively aggravated after the ischemic myocardium returns to normal perfusion (Zhao et al., 2020). Studies on the mechanism of myocardial I/R injury have been conducted for decades, and it is mainly considered to be related to cellular redox imbalance, calcium overload, ER stress, mitochondrial injury, energy depletion, and programmed cell death (Toth et al., 2007; Consolini et al., 2017; Gong et al., 2021).

An effective Ca^2+^-dependent mitochondrial energy supply is crucial for proper cardiac contractile activity, while disruption of Ca^2+^ homeostasis contributes to the pathogenesis of cardiac diseases (Gong et al., 2021). Additional evidence has suggested that several SR-mitochondria tethering complex components that regulate the calcium exchange mechanism are involved in the pathological process of I/R injury. Additionally, SR-mitochondria Ca^2+^ transfer is considered detrimental in I/R injury, for example, that of the IP3R1/GGP75/VDAC1 complex (Zhou et al., 2018a). For instance, a 2016 report showed that GSK-3β is a new Ca^2+^ regulator located in the SR/ER and MAMs, and it can specifically interact with the IP3R Ca^2+^-channeling complex. Gomez et al. (2016) reported that GSK3β inhibition diminished cytosolic and mitochondrial Ca^2+^ overload and decreased the cardiomyocyte apoptosis caused by I/R.

Recent reports have confirmed that during hypoxia-reoxygenation, Ca^2+^ overload and the excessive opening of the mPTP is an important mediator of cell death in cardiomyocytes during I/R injury (Paillard et al., 2013). It is well accepted that mitochondrial chaperone cyclophilin D (CypD), which is a component of mPTP, and accumulation of Ca^2+^ in the mitochondrial matrix can activate CypD and trigger permeability transition pore opening (Di Lisa et al., 2007). Paillard et al. (2013) found that CypD acts synergistically with the VDAC1/Grp75/IP3R1 complex, which localizes to MAMs to promote ER calcium efflux to the mitochondria. Remarkably, during H/R, this interaction is amplified with increased mitochondrial Ca^2+^ load and induces apoptosis. Inhibition of CypD or GRP75 expression and reduction of the interaction of CypD and VDAC1-IP3R1 prevented mitochondrial Ca^2+^ overload and cell death in an adult mouse cardiomyocyte H/R model (Paillard et al., 2013). Thus far, coronary microcirculation remains a difficult and neglected aspect in the treatment of myocardial I/R injury (Pries et al., 2018; Wang et al., 2020b). The latest research in 2020 demonstrated that SERCA plays an important role in this pathological process. For instance, SERCA overexpression inhibited calcium overload by regulating calcium/XO/ROS signaling and preserving the mitochondrial quality control (MQC) system to increase endothelium-dependent vasodilation, attenuate lumen stenosis, and target cardiac microvascular I/R damage (Tan et al., 2020). Likewise, overexpression of SERCA reduced reperfusion-mediated cardiac microvascular injury through inhibition of the calcium/mitochondrial calcium uniporter (MCU)/mPTP/necroptosis signaling pathways (Li et al., 2020b). These observations suggested that controlling SR-mitochondria interaction can protect cardiomyocytes against lethal reperfusion injury through the reduction of mPTP and thereby identifies new molecular targets for cardiac protection.

Excessive mitochondrial division is a predominant cause of cardiac I/R injury (Maneechote et al., 2018). Mechanistically, the Mfn1–Mfn2 heteromultimer is crucial for regulating mitochondrial fission and stabilizing ER–mitochondria microdomain formation (de Brito and Scorrano, 2008). *In vitro* studies showed that acute ablation of Mfn1 and Mfn2 (DKO) reduced the mitochondrial uptake of Ca^2+^ and decreased oxidative stress production through uncoupling the SR from mitochondria. Electron microscopy revealed predominantly fragmented interfibrillar mitochondria with loss of cristae structure in DKO intact cardiomyocytes (Papanicolaou et al., 2011; Hall et al., 2016). Importantly, the authors demonstrated that despite apparent mitochondrial dysfunction, acute deletion of Mfn1 and Mfn2 protected against cardiomyocytes I/R injury due to impaired mitochondria/ER tethering in DKO mice hearts. In conclusion, ERMCs may have beneficial roles in regulating cardiac I/R injury through modulating malignant mitochondrial fission, oxidative stress, calcium overload, and programmed cell death.

#### Endoplasmic Reticulum-Mitochondria Contacts in Atherosclerosis

Atherosclerosis is a lipid-driven chronic inflammatory disease that leads to the formation of lipid-rich and immune cell-rich plaques in the arterial intima lesions (Finney et al., 2017). The remodeling of the vascular endothelium during atherosclerosis involves alteration of the vascular cell phenotype, modulation of cell migration and proliferation, and propagation of local extracellular matrix remodeling (Perrotta, 2020).

There is evidence that the communication imbalance between the ER and mitochondria affects the function of MAMs, which leads to cardiac diseases (Safiedeen et al., 2016; Perrotta, 2020). Recent research suggested that changes in phospholipids, glucose, and key proteins located in MAMs can cause the occurrence and development of atherosclerosis (Moulis et al., 2019; Perrotta, 2020; Wang X. et al., 2021).

A 2020 study defined the role of protein p66Shc in atherosclerosis. The adaptor protein p66Shc located in the MAMs is considered as a sensor of oxidative stress-induced apoptosis (Mishra et al., 2019). Experiments with a p66Shc knockout mouse showed that a reduced number of atherosclerotic plaques formed (Napoli et al., 2003; Gil-Hernández and Silva-Palacios, 2020).

Likewise, oxidized low-density lipoprotein (ox-LDL) contributing to endothelial cell (EC) apoptosis is the first step of atherogenesis and is related to Ca^2+^ overload. The phosphofurin acid cluster classification protein 2 (PACS2) is a multifunctional tethering protein that is essential for mitochondrial Ca^2+^ overload because it mediates ER-mitochondria Ca^2+^ transfer (Yu et al., 2019). In an ox-LDL-induced apoptosis model of human umbilical vein endothelial cells (HUVECs), silencing PACS-2 inhibited ox-LDL-induced cell apoptosis and mitochondrial localization of PACS-2 and MAMs formation. Thus, this finding indicated that PACS-2 may become a promising therapeutic target for atherosclerosis by regulating MAM formation and increasing levels of mitochondrial Ca^2+^ (Yu et al., 2019).

Despite recent evidence underlining the role of ERMCs in cardiac tissue physiology, little is known about the role of ERMCs in vascular cells, including in VSMCs (Lopez-Crisosto et al., 2017; Moulis et al., 2019; Wang X. et al., 2021). However, a study published in 2019 suggested that in the VSMC apoptosis model, high-resolution confocal microscopy and adjacent ligation analysis found that the molecular changes in MAMs play a critical role in the balance between cell survival and death through modulating the interaction between autophagy and apoptosis (Moulis et al., 2019). Moulis et al. (2019) first reported that PACS-2 deletion induced MAM disruption and potentiated VSMC apoptosis. These findings suggest that MAMs-associated PACS-2 is a crucial regulator of VSMC fate during oxidized LDL-induced mitophagy. These findings reveal new insights regarding the significance of ERMCs in cell vascular pathophysiology, which indicates that manipulating ERMCs can supply new strategies to selectively improve VSMC fate and stabilize atherosclerotic plaque (Moulis et al., 2019).

As a platform to modify the phenotypic transformation of VSMCs and ECs, p66Shc and PACS-2 located on ERMCs participates in the pathological process of atherosclerosis by modifying the mitochondrial Ca^2+^ overload and apoptosis, which contributes to the survival of VSMCs and ECs. However, further research is warranted to conclusively elucidate how the molecular mechanisms in the regulation of ERMCs contribute to atherosclerosis.

## Therapy Targeting Endoplasmic Reticulum-Mitochondria Contacts for Cardiovascular Remodeling-Induced Diseases

As discussed above, alterations in the contacts between the SR/ER-mitochondria may induce cardiovascular tissue remodeling and cardiac disease. Therefore, therapeutic interventions to improve ERMCs may conserve cardiac function and represent possible promising strategies to delay cardiac aging, and to delay or prevent cardiac disease (Table 2).

**TABLE 2:** Pharmacological interventions for cardiovascular remodeling-associated diseases.

Medicine	Chemical structure	Preclinical studies	Current clinical status	References
Metformin	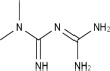	Cardiomyopathy mouse model of dystrophin-deficient; Cardiac injury in murine model during ischemia-reperfusion.	Phase IV for CHF (NCT 00473876); Phase II for CHF (NCT 03514108)	Angebault et al., 2020; Chen Q. et al., 2021
TUDCA	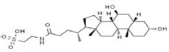	Simulated microgravity induced ER stress in rats.	Phase IV for vascular function of Type 2 DM humans (NCT 03331432); Phase II for SSA (NCT 01855360)	Zhang et al., 2020
Ibutilide	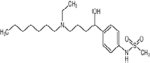	Murine model of cardiomyocytes apoptosis.	Phase I for AF (NCT 03370536)	Wang et al., 2017
Melatonin	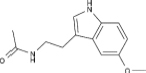	Septic cardiomyopathy in mice.	Phase II for CHF (NCT 03894683); Phase II for AMI (NCT 01172171)	Zhong et al., 2019
DFMO	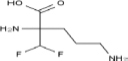	Rodent models of cardiac hypertrophy.	NA	Zhao et al., 2021

*TUDCA, tauroursodeoxycholic acid; I/R, ischemia-reperfusion; CHF, chronic heart failure; DFMO, difluoromethylornithine; DM, diabetes mellitus; SSA, senile systemic amyloidosis; AMI, acute myocardial infarction.*

### Pharmacological Interventions

One option to improve the ER–mitochondrial contacts is by means of metformin, a traditional anti-diabetic drug with pleiotropic properties associated with its mitochondrial effects (Pries et al., 2018). Administration of metformin decreased MICU1 expression and mitochondrial Ca^2+^ content, and enhanced complex I-driven respiration in a cardiomyopathy mouse model of dystrophin-deficiency, suggesting that metformin has a cardioprotective role in impaired Ca^2+^ homeostasis and aberrant SR/ER-mitochondria interaction (Angebault et al., 2020). Moreover, metformin decreased the infarct size during cardiac I/R through attenuating CHOP expression and subsequently protecting the mitochondria (Chen Q. et al., 2021).

In a phase IV randomized controlled trial (NCT 00473876) with metformin in patients with chronic heart failure, it was reported that metformin treatment significantly improved the VE/VCO_2_ slope but had no effect on peak VO_2_, which reflected exercise capacity (Das et al., 2019). A phase II randomized, placebo-controlled study in chronic heart failure (CHF) patients with metformin (NCT 03514108), which was designed to determine whether metformin reduces the incidence of worsening heart failure and acute myocardial infarction in patients with diabetes, is still in progress (Wiggers et al., 2021).

Another option is the utilization of tauroursodeoxycholic acid (TUDCA) and 4-phenylbutyric acid (4-PBA), which are ER stress inhibitors. This proved to be beneficial in ameliorating mitochondrial oxidative injury and ER stress-mediated cerebrovascular VSMC phenotypic transition through the PERK-eIF2a-A TF4-CHOP pathway in a rat model. A randomized, placebo-controlled study of type 2 diabetes mellitus patients with TUDCA (NCT 03331432) aimed to evaluate the effect of TUDCA administration on enhancement of vascular function in humans, and the trial is currently still in progress (Wiggers et al., 2021). Additionally, a phase II open label trial with TUDCA (NCT 01855360), which was designed to evaluate the safety and tolerability of the combination of TUDCA and doxycycline in familial and senile amyloidosis patients, has not yet been completed.

Moreover, in H_2_O_2_-induced apoptosis of neonatal rat cardiomyocytes, ibutilide, a class III antiarrhythmic agent, attenuated ER stress (GRP78, GRP94e, and CHOP), mitochondrial oxidative stress, and mitochondrial-dependent apoptosis (Bax, Bcl-2, and caspase-3/9/12) (Wang et al., 2017). A prospective, non-randomized, un-blinded, observational trial (NCT 03370536) investigating the effectiveness of ibutilide on atrial fibrillation (AF) source location and organization is under advanced development.

The therapeutic contribution of melatonin reversed changes in mitochondrial dynamics, ameliorated ER stress, and prevented the disassembly of the cardiomyocyte cytoskeleton by repressing RIPK3 in a septic cardiomyopathy mouse model. This study showed that melatonin simultaneously modulated mitochondrial homeostasis and ER function (Zhong et al., 2019). In brief, this finding highlights the role of melatonin, which may be considered as an ideal agent for the targeted therapy of septic cardiomyopathy via modulating mitochondrial homeostasis and ER function. An attempt at therapy in a randomized placebo-controlled phase II trial (NCT 03894683) is currently ongoing to determine the effect of melatonin on cardiovascular and muscle mass and function in patients with CHF (Sadeghi et al., 2020). Another randomized, placebo-controlled trial (NCT 01172171), which was designed to determine whether administration of melatonin reduces infarct size in patients with acute myocardial infarction, is proceeding (Dominguez-Rodriguez et al., 2007). If successful, these findings would support the therapeutic use of melatonin for cardiac disease.

Finally, in a rat model of cardiac hypertrophy induced by isoproterenol, administration of difluoromethylornithine (DFMO) attenuated cardiac hypertrophy through downregulating the MAM signaling pathway (cleaved caspase-3/9, GRP75, Mfn2, CypD, and VDAC1) and upregulating the autophagy pathway in heart tissue (Zhao et al., 2021). These findings suggested that DFMO treatment could provide a potential strategy for preventing ISO-induced cardiac hypertrophy.

Several clinical medicines may improve ERMCs, thereby having a beneficial effect on cardiovascular remodeling-induced diseases. However, further research is required to elucidate compounds that directly target the ERMCs (Li et al., 2019).

### Lifestyle Interventions

Lifestyle change consisting of regular physical activity and caloric restriction (CR) demonstrated protection against cardiac risk factors and improved cardiorespiratory fitness in humans (Makar and Siabrenko, 2018; Kumar et al., 2020).

In peripheral blood mononuclear cells (PBMCs) from healthy elderly subjects, 8-week resistance-training (RT) exercise-induced UPR activation counteracted ERS and led to mitochondrial improvement and prevention of mitophagy activation through increased ATF4, XBP1, PGC-1α, and Mfn1 protein levels (Estebanez et al., 2019). The results showed that RT resulted in the UPR, as a protective mechanism, and mitochondrial biogenesis to adapt to the exercise demands. In addition, in a diabetic mouse model, it was found that high-intensity training improved cardiac function and reduced cardiac infarction by downregulating GRP78, phosphorylated PERK, phosphorylated eIF2α, ATF4, ATF6, XBP1, CHOP, and cleaved caspase-3 (Bourdier et al., 2016; Bozi et al., 2016).

Finally, a study suggested that an 8-week diet reversal alleviated the T2D-induced cardiac dysfunction via reestablishing the functional Ca^2+^ coupling of the ER–mitochondria interface and a normal Ca^2+^ transfer in the high-fat high-sucrose diet (HFHSD)-induced obesogenic T2D mouse model (Dia et al., 2020).

The impact of lifestyle interventions on ERMCs modification remains an open question (Li et al., 2019; Kumar et al., 2020; Sun and Ding, 2020). Much input is required to understand whether and how alterations in ERMCs are involved in cardiovascular remodeling-induced disease enhancement, and how different types of lifestyle interventions counteract this.

## Conclusion

As outlined in this review, cardiovascular disease caused by cardiovascular remodeling is still the main cause of the disease incidence rate and mortality worldwide. An increasing amount of evidence has shown that ERMCs play an important role in the pathologic process of cardiovascular remodeling. The functional balance and interaction between the ER and mitochondria are prerequisites for a healthy heart and optimal vascular function, and loss of this interaction can exacerbate cardiovascular remodeling and cardiovascular disease.

Here, we describe the concept, structure, and function of the interaction between the ER and mitochondria, and discuss how the imbalance of interaction exacerbates cardiovascular diseases such as heart failure and atherosclerosis. However, more studies are required to define the role that alterations in ERMCs have in the pathogenesis of cardiovascular remodeling-associated diseases (Li et al., 2019). Obviously, due to the different substrates and mechanisms of the energy metabolism of cardiomyocytes, vascular ECs, and VSMCs, the effects of ERMCs in these cell types are not identical (Gao et al., 2020).

The relationship between ERMCs regulation and cardiovascular remodeling is two-sided. The journey that calcium must take between the ER and its mitochondrial destination requires several regulatory steps and molecular checkpoints, and any alterations result in dramatic metabolic or apoptotic defects. However, any increase in ER-mitochondrial proximity enhances mitochondrial Ca^2+^ uptake, thus activating an excitation-contraction coupling process and ATP synthesis of cardiomyocytes (Safiedeen et al., 2016; Marchi et al., 2018). Therefore, a deeper understanding and precise regulation of ERMCs may be a promising method to develop more specific treatment strategies and selectively inhibit the progression of remodeling. Finally, we enumerated some drugs: Metformin, TUDCA, Ibutilide, Melatonin and DFMO, which have the potential to preserve this ER-mitochondrial interaction, but also need further study to identify the exactly effect on ERMCs. Further well-designed clinical trials are required to explore the underlying mechanism and prove their efficacy in cardiovascular remodeling-induced diseases.

## Author Contributions

CL, XL, and YuW conceived the project. CL, YuW, XZ, YaW, and RX wrote the manuscript. XZ and RX prepared the figure. SL finished the table. All authors have reviewed the manuscript.

## Conflict of Interest

The authors declare that the research was conducted in the absence of any commercial or financial relationships that could be construed as a potential conflict of interest.

## Publisher’s Note

All claims expressed in this article are solely those of the authors and do not necessarily represent those of their affiliated organizations, or those of the publisher, the editors and the reviewers. Any product that may be evaluated in this article, or claim that may be made by its manufacturer, is not guaranteed or endorsed by the publisher.
